# Preparedness for generative AI adoption among Chinese cancer survivors: a multi-center cross-sectional survey study

**DOI:** 10.3389/fpubh.2026.1779200

**Published:** 2026-05-18

**Authors:** Xin Wang, Linjuan Li, Xuebin Cai, Wenjing Luo, Mengdie Shen, Yan Zuo, Jianjun Zhang, Zhilan Bai, Yutang Yao, Musi Zhang, Ying Zhang

**Affiliations:** 1 Division of Abdominal Tumor Multimodality Treatment, Cancer Center, West China Hospital, Sichuan University/West China School of Nursing, Sichuan University, Chengdu, China; 2 Thoracic Oncology Ward, Cancer Center, West China Hospital, Sichuan University/West China School of Nursing, Sichuan University, Chengdu, China; 3 Department of Gynecology and Obstetrics Nursing, West China Second University Hospital, Sichuan University/West China School of Nursing, Sichuan University, Chengdu, Sichuan, China; 4 Key Laboratory of Birth Defects and Related Disease of Women and Children, Ministry of Education, Sichuan University, Chengdu, Sichuan, China; 5 Department of Gynecology and Obstetrics, West China Second University Hospital, Sichuan University/Key Laboratory of Birth Defects and Related Diseases of Women and Children, Ministry of Education, Sichuan University, Chengdu, China; 6 Department of Nuclear Medicine, Sichuan Cancer Hospital and Institute, Sichuan Cancer Center, University of Electronic Science and Technology of China, Chengdu, China; 7 Department of Radiation Oncology, Sichuan Cancer Hospital and Institute, Sichuan Cancer Center, University of Electronic Science and Technology of China, Chengdu, China; 8 Division of Internal Medicine, Institute of Integrated Traditional Chinese and Western Medicine, West China Hospital, Sichuan University, Chengdu, China

**Keywords:** adoption preparedness, cancer survivorship, digital health literacy, generative artificial intelligence, health information ability, large language model, technology acceptance

## Abstract

**Background:**

Generative artificial intelligence (GenAI) is rapidly entering consumer health information environments, yet patient readiness for safe adoption in cancer survivorship remains unclear. This study assessed preparedness for GenAI adoption among Chinese cancer survivors and identified correlates relevant to equitable implementation.

**Methods:**

We conducted a multi-center, cross-sectional online survey among digitally reachable adult cancer survivors recruited via clinical encounters, WeChat patient groups, and peer referral across three oncology centers in Sichuan, China. The questionnaire was administered on Wenjuanxing. The primary outcome was a theory-informed, study-specific 0–100 GenAI adoption preparedness composite derived from five Likert items: perceived usefulness, perceived ease of use, access to guidance/support, privacy concern after reverse coding, and near-term intention. Secondary outcomes included GenAI awareness and prior use, willingness for report explanation and symptom advice scenarios, and health information ability. Multivariable linear regression with robust standard errors estimated adjusted associations with preparedness, with sensitivity analyses addressing data quality flags and recruitment pathway.

**Results:**

From 1,062 survey visits, 876 participants comprised the analytic sample. Mean preparedness was 57.8 (SD 24.2) with acceptable internal consistency (Cronbach’s alpha 0.75). Awareness of GenAI was 61.6 and 40.6% reported prior use. Near-term intention to try GenAI for survivorship information tasks was endorsed by 51.3%. Willingness was higher for test report explanation (56.1% agree/strongly agree) than for symptom advice with referral prompts (40.3%). Preparedness was lower among participants older than 60 years versus 18–45 years (beta −8.1, 95% CI −12.0 to −4.3) and higher with prior generative AI use (beta 9.3, 95% CI 5.7–12.9), higher self-rated generative AI knowledge (beta 5.4 per 1-point, 95% CI 3.7–7.0), and greater health information ability (beta 2.0 per 10 points, 95% CI 1.2–2.7). The model explained 36% of variance (R^2^ 0.36).

**Conclusion:**

Among digitally reachable cancer survivors in Sichuan, preparedness for GenAI adoption was moderate and strongly use-case dependent, with lower readiness among older survivors. The online-only sampling strategy means that the observed preparedness level may overestimate readiness in the broader survivorship population. Implementation should begin with lower-risk applications, such as report explanation and question preparation, paired with guidance on verification, privacy protection, and clear escalation to clinicians.

## 1 Introduction

Cancer survivorship has become a central public health challenge in China as population aging, rising cancer incidence, and improving survival have produced a growing population living with the long-term consequences of cancer and its treatment (1). Recent national estimates indicate a substantial cancer burden, with roughly 4.8 million new cancer cases and 2.6 million deaths in China in 2022, and a disease profile dominated by lung, colorectal, thyroid, liver, and stomach cancers (2–5). At the same time, survival in China has improved across many cancer types, reflected in multicenter evidence reporting an age-standardized 5-year relative survival near the mid-40% range in 2019–2021, albeit with wide heterogeneity by cancer type (6). As survivorship expands, the needs of survivors extend beyond surveillance for recurrence to include symptom management, treatment sequelae, lifestyle modification, psychosocial adaptation, medication adherence, and navigation of fragmented care pathways (7). The informational and self-management demands of survivorship are therefore frequent, recurrent, and unevenly met, with risk of widening disparities for older adults, individuals with lower education, and residents outside high-resource urban settings (8).

Digital channels have already become a major route for cancer-related information seeking and support (9). In China, qualitative work in cancer patients has highlighted ongoing information needs, preferences for culturally compatible communication, and the influence of social and relational contexts on information seeking behaviors (10). Complementing this, recent scholarship has examined online health information seeking patterns among Chinese breast cancer populations and has underscored variation by individual characteristics, reinforcing the likelihood that the benefits of digital information are distributed unevenly across subgroups (11, 12). In parallel, nationally oriented analyses of cancer survivorship in China have linked health information acquisition to downstream behavioral and psychosocial factors, suggesting that information environments and trust-related constructs matter for survivorship outcomes (13, 14). Collectively, available evidence supports a practical conclusion: cancer survivors in China already use digital tools to fill informational gaps, but the ability to locate, interpret, and act on health information remains variable, creating both opportunity and vulnerability when novel information technologies become widely accessible (15).

Generative artificial intelligence (GenAI), particularly large language model (LLM) chatbots, has recently emerged as a potentially transformative layer within consumer health information ecosystems (16). Compared with conventional search, GenAI tools can generate natural-language explanations, summarize complex text, translate between technical and lay language, and support preparation for clinical encounters by drafting questions and organizing symptom histories (16). In consumer health education contexts, recent reviews have described expanding use cases while also emphasizing the need for clinician-informed evaluation frameworks that prioritize safety, usability, and effectiveness (17, 18). In oncology specifically, published reviews have summarized early evidence suggesting that LLMs can produce apparently useful cancer-related information for screening or management questions, while also demonstrating non-trivial error rates and susceptibility to obsolete or unsupported content (19–21). Cross-language evaluation work has similarly demonstrated that publicly accessible LLMs can generate plausible cancer-related answers across multiple languages, reinforcing both promise and risk in real-world multilingual deployment (22, 23).

For cancer survivorship, the public health relevance of GenAI is shaped by a tension between access and safety (24). On the opportunity side, GenAI may reduce literacy barriers by providing iterative explanations, tailoring the level of detail, and supporting question formulation for time-limited consultations (25). Such capabilities could be particularly meaningful in survivorship, where uncertainty is common and information needs recur across follow-up visits (26). On the risk side, GenAI systems can produce “hallucinations,” meaning confident statements that are factually incorrect or not supported by reliable evidence, and may fail silently in ways that are difficult for lay users to detect (27, 28). Reviews focused on patient safety have emphasized that foundation models introduce new risks and unknowns alongside potential benefits, including misinformation, automation bias, and unclear accountability pathways (29, 30). Regulatory and governance discussions have further highlighted that LLM-based health applications can expose patients to unverified medical advice in settings where oversight may be ambiguous, adding urgency to risk-mitigation frameworks and safe-use guidance (31, 32). Privacy risk is also salient because GenAI interactions can involve sensitive medical information, and data-intensive model development and deployment can introduce threats to protected health information if privacy safeguards are unclear or weak (33). For cancer survivors, who may already face stigma and discrimination concerns, privacy anxiety may meaningfully constrain willingness to adopt patient-facing GenAI, even when perceived usefulness is high (34).

Because GenAI tools are entering everyday life through mainstream platforms, the question is no longer only whether GenAI can support health tasks in principle, but also whether intended users are prepared to adopt such tools safely and equitably (35). Preparedness for GenAI adoption can be conceptualized as an implementation-relevant readiness construct that integrates perceived usefulness, ease of use, and facilitating conditions with health-specific modifiers such as perceived risk of harm, trust, and privacy concern (36). Technology acceptance models provide a structured basis for measuring such readiness. The Unified Theory of Acceptance and Use of Technology (UTAUT) posits performance expectancy, effort expectancy, social influence, and facilitating conditions as core determinants of behavioral intention and use, with UTAUT2 extending the model for consumer contexts and encouraging context-specific extensions (37, 38). Within health contexts, extensions that incorporate trust, perceived risk, and privacy concern are particularly defensible because adoption decisions carry perceived stakes beyond convenience and productivity (39, 40). Recent GenAI research in other domains has similarly identified hallucinations and privacy concerns as central risks shaping continued use, reinforcing the plausibility that safety and privacy perceptions are not peripheral but central to adoption readiness (41, 42).

Despite rapidly expanding commentary and evaluations of LLM performance in health tasks, evidence remains limited regarding GenAI preparedness among cancer survivors in China. Existing work has contributed important insights into cancer-related online information seeking in Chinese contexts, but most studies predate widespread public use of LLM chatbots or focus on internet searching rather than conversational generation (43, 44). Meanwhile, emerging qualitative evidence indicates that Chinese oncology professionals have nuanced attitudes about endorsing AI-driven chatbots for patient information seeking, suggesting that real-world integration will hinge on both clinician endorsement and patient readiness (45, 46). A survivorship-focused readiness assessment is therefore timely because GenAI tools may intensify existing inequities if adoption is concentrated among younger, more educated, digitally skilled subgroups, while vulnerable survivors remain excluded or exposed to higher risk through low digital health capability.

Against this background, the present study aimed to characterize preparedness for GenAI adoption among Chinese cancer survivors using a theoretical framework-supported questionnaire administered online via Wenjuanxing and disseminated through clinician-facilitated recruitment and patient referral routes. Preparedness was operationalized as a composite reflecting perceived usefulness, perceived ease of use, availability of guidance for safe use, privacy concern, and near-term intention to try GenAI for survivorship-related information tasks. Secondary objectives were to describe GenAI awareness and prior use, quantify willingness to use GenAI in two practical survivorship scenarios (report interpretation and symptom advice accompanied by referral prompts), and evaluate associations of preparedness with sociodemographic, clinical, digital access, GenAI knowledge, and health information ability factors. The study was designed to support public health decision-making by identifying subgroups with lower readiness and by highlighting modifiable barriers that can inform patient education, clinician guidance, and safer implementation strategies.

The contribution of this study lies in providing an early, survivorship-specific readiness baseline for GenAI adoption in a large Chinese sample, using a transparent, theory-informed measurement approach that explicitly incorporates privacy concern and safety-relevant considerations. By linking preparedness to health information ability and GenAI exposure, the findings can inform targeted interventions that improve equitable access to safe GenAI use, guide clinicians and health systems on realistic support needs, and shape risk-mitigation priorities for patient-facing GenAI deployment in survivorship care.

## 2 Methods

### 2.1 Study design, setting, and reporting framework

A multi-center, cross-sectional survey study was conducted to characterize preparedness for adopting GenAI among adult cancer survivors who could be reached through clinical and online patient-referral channels. Recruitment was implemented through three participating oncology centers in Sichuan Province, China: West China Hospital, West China Second University Hospital, and Sichuan Cancer Center. Because the survey relied on convenience and participant referral mechanisms, and the questionnaire did not capture center identifiers in a way that reliably attributed responses to a single center, analyses were prespecified and conducted on the pooled sample without center-stratified reporting or site-level random effects. This design choice was treated as an analytic limitation rather than as evidence that center-level heterogeneity was absent.

### 2.2 Participants

Participants were eligible if they were aged 18 years or older, self-reported a history of physician-diagnosed malignant tumor (cancer), were able to read and respond to a Mandarin Chinese questionnaire, and provided electronic informed consent at the beginning of the survey. Respondents were excluded if they did not consent, reported no cancer diagnosis or uncertainty about the diagnosis at screening, or reported being younger than 18 years.

### 2.3 Sampling strategy and recruitment procedures

A non-probability sampling strategy combining convenience and snowball sampling was used. Convenience sampling occurred when oncology nurses and physicians introduced the study to potential participants during routine clinical encounters and provided the survey access QR code. In parallel, the survey QR code was distributed through WeChat patient groups associated with the participating centers. Snowball sampling was facilitated by encouraging participants to share the QR code with fellow patients who might meet eligibility criteria. Because all survey completion occurred online and 99.0% of the analytic sample reported access to an internet-enabled smartphone, the recruited sample was interpreted as a digitally reachable survivor sample rather than as a representative sample of all cancer survivors in China. Recruitment route was recorded for transparency and sensitivity analyses by recruitment pathway, but center-specific attribution was not used in reporting.

### 2.4 Survey platform, administration, and consent

The questionnaire was administered online only using Wenjuanxing. The survey was accessible via QR code using a smartphone browser or embedded WeChat browser. Informed consent was embedded on the first page of the questionnaire, and respondents could proceed only after selecting the consent option. The survey was designed to be completed within 5–10 min to minimize respondent burden. Adaptive logic was used so that items on frequency and purposes of GenAI use were presented only to respondents reporting prior GenAI use. Most items were configured as required fields to minimize missingness for primary and secondary outcomes.

### 2.5 Questionnaire development and content

The instrument was theoretical framework-supported and developed specifically for this survivorship survey rather than taken from a single previously validated GenAI preparedness scale. Item content was informed by technology acceptance theory, health information behavior literature, and patient-safety concerns specific to consumer GenAI. Draft items were reviewed by oncology nursing, clinical oncology, and digital health research team members for content relevance, clarity, and respondent burden, then refined after informal pretesting for plain-language comprehension and survey flow. Prior to GenAI-related items, the questionnaire defined GenAI using plain-language examples relevant to Chinese users, including Kimi, Tongyi Qianwen, Wenxin Yiyan, and iFLYTEK Spark, and stated that GenAI outputs might be inaccurate and cannot replace clinicians. The final survey comprised 30 questions across the following domains: recruitment pathway and eligibility screening; demographics and clinical context; digital access; GenAI awareness and use; health information ability; an attention check; GenAI adoption preparedness; and willingness to use GenAI in two survivorship-related scenarios. Items for health information ability and preparedness were rated on 5-point Likert scales. Scenario willingness items presented short vignettes and asked about willingness using the same 5-point scale.

### 2.6 Outcomes and scoring rules

The primary outcome was a GenAI adoption preparedness score computed from five preparedness items capturing perceived usefulness, perceived ease of use, access to guidance/support, privacy concern, and near-term intention to try GenAI. The composite was used as a theory-informed implementation-readiness measure, not as a fully validated psychometric scale. Perceived usefulness and ease of use represented core acceptance dimensions, access to guidance/support represented facilitating conditions for safe use, privacy concern represented a health-specific barrier to adoption, and near-term intention represented the behavioral-readiness component of preparedness. Privacy concern was reverse coded so that higher values consistently indicated higher preparedness. Near-term intention was retained in the composite because the study focused on adoption preparedness rather than abstract attitudes alone; however, it was also reported separately as a descriptive secondary outcome to make its role transparent. The preparedness score was calculated as the mean of the five items and linearly transformed to a 0–100 scale using (mean − 1) × 25, where higher scores indicate greater preparedness.

Secondary outcomes included near-term intention to try GenAI, dichotomized as agree or strongly agree versus other responses for descriptive reporting; willingness to use GenAI for report explanation and for symptom advice with referral prompts, each dichotomized similarly for descriptive reporting; and descriptive measures of GenAI awareness and prior use. A health information ability index was computed as the mean of three Likert items reflecting perceived ability to locate health information, judge online information reliability, and preference to consult clinicians for major health decisions. The third item was retained because safe use of GenAI in oncology depends not only on locating and appraising information but also on recognizing when major decisions should be anchored in clinician advice. The index was interpreted as a pragmatic information appraisal and clinical help-seeking orientation measure, and was transformed to a 0–100 scale for interpretability.

### 2.7 Data quality control

Quality control procedures were specified to align with online-only administration. An attention check item was placed immediately after the health information ability items. Respondents who did not select the instructed response were flagged as potentially low-quality but were not automatically excluded from the primary analysis. Completion time was recorded; submissions completed in under 180 s were flagged as unusually rapid. Potential duplicate submissions were assessed using Wenjuanxing metadata, where available, together with submission timing and response similarity. Records were treated as suspected duplicates when they shared device or browser metadata or an internet protocol address, had closely adjacent submission times, and showed highly similar responses across eligibility, demographic, and preparedness items; when duplicates were strongly suspected, only the first valid record was retained based on a prespecified rule. Sensitivity analyses were planned to evaluate robustness after excluding attention-check failures, unusually rapid submissions, and, where possible, after adjustment for recruitment pathway.

### 2.8 Statistical analysis

Participant characteristics and survey measures were summarized using counts and percentages for categorical variables and means with standard deviations for continuous variables. Internal consistency of the preparedness scale was evaluated using Cronbach’s alpha after aligning item directionality via reverse coding of privacy concern. Item coherence was further assessed descriptively by examining item distributions and item-total behavior. The primary analysis examined correlates of preparedness using multivariable linear regression with the 0–100 preparedness score as the dependent variable. Independent variables included age group, sex, residence, education, time since diagnosis, treatment status, daily internet use time, frequency of using the internet to obtain health information, self-rated GenAI knowledge, prior GenAI use, self-rated health, and health information ability. Robust heteroskedasticity-consistent standard errors were used. Multicollinearity was assessed using variance inflation factors, and no variable was retained if it showed problematic collinearity with conceptually overlapping predictors. Continuous predictors were inspected for approximately linear associations with preparedness, and influential observations were evaluated using standard regression diagnostics. Reference categories were prespecified for interpretability. Sensitivity analyses repeated the primary model after excluding respondents flagged by the attention check or rapid completion time and after considering recruitment pathway. Statistical analyses were conducted using standard statistical software, with two-sided significance testing at *α* = 0.05.

### 2.9 Ethics

Electronic informed consent was obtained at the start of the online questionnaire. The study protocol was approved by the Ethics Committees of West China Hospital, Sichuan University (approval number 2025-2193). The survey did not collect direct personal identifiers such as names or national identification numbers.

## 3 Results

A total of 1,062 unique visits to the survey landing page were recorded during the recruitment period. Among these, 969 respondents provided electronic consent and entered the questionnaire. Forty-five respondents were screened out due to age under 18 years or reporting no or uncertain cancer diagnosis, leaving 924 eligible respondents. Of eligible respondents, 905 submitted the questionnaire, corresponding to a completion rate of 97.9% among eligible participants. After removal of 29 records because of suspected duplicate submission or missing primary preparedness items, the analytic sample included 876 participants. The exact breakdown between suspected duplicate records and missing-outcome exclusions should be verified against the locked survey export before final submission.

Median completion time was 423 s (interquartile range 232–542 s). Fifty-six respondents (6.4%) failed the attention check, and nine respondents (1.0%) completed the survey in under 180 s. These respondents were retained in the primary analyses and examined in prespecified sensitivity analyses because rapid completion or attention-check failure could indicate lower response quality but did not prove invalid eligibility or outcome data.

### 3.1 Participant characteristics

The analytic sample (*n* = 876) included 327 participants (37.3%) aged 18–45 years, 335 (38.2%) aged 45–60 years, and 214 (24.4%) aged older than 60 years. Females comprised 54.2% of the sample, and 65.3% reported urban residence. Breast cancer (23.6%), lung cancer (16.3%), and colorectal cancer (13.2%) were the most commonly reported cancer types. The largest subgroup by treatment status was post-operative recovery or follow-up (42.8%), and 31.5% reported currently receiving systemic therapy. The mean self-rated health score was 3.13 (SD 1.25) on a 1–5 scale. Participant characteristics are shown in Table 1.

**Table 1:** Participant characteristics (analytic sample, *n* = 876).

Characteristic	Category	*n* (%)
Age group (years)	18–45	327 (37.3)
	45–60	335 (38.2)
	>60	214 (24.4)
Sex	Male	389 (44.4)
	Female	475 (54.2)
	Prefer not to say	12 (1.4)
Residence	Urban	572 (65.3)
	Rural	288 (32.9)
	Uncertain/Prefer not to say	16 (1.8)
Education	Primary or below	96 (11.0)
	Junior secondary	173 (19.7)
	Senior secondary/Technical	188 (21.5)
	College	131 (15.0)
	Bachelor	174 (19.9)
	Postgraduate or over	97 (11.1)
	Prefer not to say	17 (1.9)
Cancer type	Breast	207 (23.6)
	Lung	143 (16.3)
	Colorectal	116 (13.2)
	Gastric	84 (9.6)
	Liver	73 (8.3)
	Gynecologic	75 (8.6)
	Hematologic	51 (5.8)
	Other	84 (9.6)
	Uncertain/Prefer not to say	43 (4.9)
Time since diagnosis	<6 months	170 (19.4)
	6–12 months	150 (17.1)
	1–3 years	236 (26.9)
	3–5 years	141 (16.1)
	>5 years	144 (16.4)
	Uncertain/Prefer not to say	35 (4.0)
Current treatment status	Systemic therapy	276 (31.5)
	Radiotherapy	65 (7.4)
	Post-operative recovery/follow-up	375 (42.8)
	Recurrence/metastasis treatment	108 (12.3)
	Uncertain/Prefer not to say	52 (5.9)
Self-rated health (1–5)	Mean (SD)	3.13 (1.25)

### 3.2 Digital access and GenAI exposure

Digital access was high in this online-only sample, with 99.0% reporting an internet-enabled smartphone. Daily internet use of at least 2 h was reported by 54.0, and 48.5% reported often using the internet to obtain health information. Awareness of GenAI prior to the survey was reported by 61.6, and 40.6% reported having ever used GenAI. Self-rated GenAI knowledge averaged 3.55 (SD 1.15) on a 1–5 scale. Among respondents reporting prior GenAI use (*n* = 356), the most common usage frequency in the past 30 days was 1–3 times per month (39.0%), followed by 1–3 times per week (34.6%). The most frequently reported purposes of GenAI use included daily life queries (56.5%), health information seeking (54.5%), and work or study writing (48.6%). Detailed measures of digital access and GenAI exposure are presented in Table 2.

**Table 2:** Digital access and GenAI awareness/use (*n* = 876; among ever users *n* = 356).

Measure	Category	*n* (%)
Has internet-enabled smartphone	Yes	867 (99.0)
	No	9 (1.0)
Daily internet use time	<1 h/day	129 (14.7)
	1–2 h/day	253 (28.9)
	2–4 h/day	315 (36.0)
	>4 h/day	158 (18.0)
	Uncertain/Prefer not to say	21 (2.4)
Uses internet to obtain health information	Often	425 (48.5)
	Sometimes	338 (38.6)
	Rarely/Never	113 (12.9)
Heard of GenAI before the survey	Yes	540 (61.6)
	No	336 (38.4)
Ever used GenAI	Yes	356 (40.6)
	No	520 (59.4)
Self-rated GenAI knowledge (1–5)	Mean (SD)	3.55 (1.15)
Among ever users (*n* = 356)	Past-30-day GenAI use frequency	
GenAI use frequency (past 30 days)	Almost daily	31 (8.7)
	1–3 times/week	123 (34.6)
	1–3 times/month	139 (39.0)
	Not in past 30 days	52 (14.6)
	Uncertain	11 (3.1)
Among ever users (*n* = 356)	Primary purposes (multi-select)	
GenAI purposes (multi-select)	Work/study writing	173 (48.6)
	Daily life queries	201 (56.5)
	Health information	194 (54.5)
	Emotional support/chat	84 (23.6)
	Other	43 (12.1)

### 3.3 Health information ability, GenAI preparedness, and scenario willingness

Health information ability averaged 63.8 (SD 20.4) on the 0–100 scale. The primary outcome, GenAI adoption preparedness, averaged 57.8 (SD 24.2) on the 0–100 scale. The preparedness scale showed acceptable internal consistency after aligning item directionality, with Cronbach’s alpha of 0.75. Item-level results indicated that perceived usefulness and ease of use were higher than perceived access to guidance/support, while privacy concern was moderate. The health information ability items showed the expected pattern of moderate perceived information-location and appraisal ability alongside stronger preference for clinician consultation in major decisions, supporting interpretation of the index as a pragmatic appraisal and help-seeking orientation measure rather than a pure digital literacy scale. In terms of near-term intention, 51.3% agreed or strongly agreed that they would try GenAI for health information tasks in the next 3 months if an appropriate tool were available. Willingness was higher for the report explanation scenario than for the symptom advice scenario. Specifically, 56.1% agreed or strongly agreed they would use GenAI for explaining test reports, while 40.3% agreed or strongly agreed they would use GenAI for symptom advice even when paired with referral prompts. Full scale and scenario results are shown in Table 3.

**Table 3:** Scale performance and main outcomes (*n* = 876).

Domain	Measure	Value	Additional
Health information ability items (1–5)	Find needed health information (Q20)	3.41 (1.26)	
	Judge reliability of online health information (Q21)	3.39 (1.28)	
	Prefer consulting clinicians for major health decisions (Q22)	3.85 (0.92)	
Health information ability score	Mean of Q20-Q22	3.55 (0.82)	
	0–100 transformed	63.8 (20.4)	
GenAI adoption preparedness items (1–5)	Perceived usefulness (Q24)	3.76 (1.28)	
	Perceived ease of use (Q25)	3.54 (1.34)	
	Access to guidance/support (Q26)	3.29 (1.36)	
	Privacy concern (Q27; higher = more concern)	3.21 (0.95)	
	Intention to try in next 3 months (Q28)	3.41 (1.40)	51.3% ≥4
GenAI adoption preparedness score	Mean of Q24-Q28 after reversing Q27	3.31 (0.97)	Cronbach’s α = 0.75
	0–100 transformed	57.8 (24.2)	
Scenario willingness (1–5)	Report explanation scenario (Q29)	3.54 (1.45)	56.1% ≥4
	Symptom advice with referral prompts (Q30)	2.98 (1.53)	40.3% ≥4

### 3.4 Factors associated with GenAI adoption preparedness

Preparedness varied meaningfully by age group and GenAI experience. Mean preparedness was 64.3 (SD 21.4) among respondents aged 18–45 years, 58.0 (SD 24.3) among those aged 45–60 years, and 47.6 (SD 24.5) among those older than 60 years. Preparedness was substantially higher among respondents who had ever used GenAI (mean 71.0, SD 18.9) compared with those who had not (mean 48.8, SD 23.2).

In multivariable linear regression adjusting for demographic, clinical, and digital covariates, older age remained associated with lower preparedness, with respondents older than 60 scoring 8.1 points lower on the 0–100 preparedness scale than those aged 18–45. Greater health information ability was independently associated with higher preparedness, with a 10-point increase in the health information ability index corresponding to a 2.0-point higher preparedness score. Prior GenAI use and higher self-rated GenAI knowledge were among the strongest correlates, with ever users scoring 9.3 points higher than never users and each 1-point increase in GenAI knowledge associated with a 5.4-point higher preparedness score. Time since diagnosis of 3–5 years was associated with slightly lower preparedness compared with 1–3 years. Other covariates, including sex, residence, and most education categories, were not independently associated with preparedness in the adjusted model. The model explained 36% of variance in preparedness (*R*^2^ = 0.36). Full regression results are shown in Table 4.

**Table 4:** Multivariable linear regression for preparedness (0–100 outcome, *n* = 876).

Predictor	Category	Beta	95% CI	*p*
Age group	45–60	−2.6	−5.7–0.6	0.109
	>60	−8.1	−12.0 to −4.3	<0.001
Sex	Female	1.8	−0.9–4.6	0.185
	Other/Prefer not to say	−8.5	−19.9–3.0	0.149
Residence	Rural	−2.2	−5.1–0.7	0.143
	Uncertain/Prefer not to say	−1.7	−10.4–6.9	0.694
Education	Primary or below	−3.1	−8.5–2.4	0.271
	Junior secondary	0.2	−4.2–4.7	0.919
	College	0.2	−4.4–4.8	0.929
	Bachelor	−2.1	−6.3–2.1	0.324
	Postgraduate+	−3.8	−8.9–1.3	0.143
	Prefer not to say	−1.2	−11.2–8.8	0.813
Time since diagnosis	<6 months	0.5	−3.7–4.6	0.824
	6–12 months	1.4	−2.6–5.4	0.494
	3–5 years	−5.1	−9.4 to −0.9	0.018
	>5 years	−0.4	−4.7–3.9	0.849
	Uncertain/Prefer not to say	−1	−8.8–6.8	0.793
Treatment status	Systemic therapy	−1.5	−4.7–1.7	0.346
	Radiotherapy	2.1	−3.5–7.7	0.463
	Recurrence/metastasis treatment	0.5	−3.9–5.0	0.817
	Uncertain/Prefer not to say	3.4	−1.9–8.7	0.204
Daily internet use time	<1 h/day	−0.9	−5.2–3.4	0.671
	2–4 h/day	−0.6	−3.9–2.7	0.727
	>4 h/day	−0.7	−4.9–3.5	0.754
	Uncertain/Prefer not to say	2.8	−7.7–13.3	0.596
Uses internet for health information	Often	2.5	−0.3–5.4	0.084
	Rarely/Never	−3.4	−7.7–0.9	0.122
Self-rated health (1–5)	Per 1-point increase	0.6	−0.5–1.7	0.281
Ever used GenAI	Yes vs. No	9.3	5.7–12.9	<0.001
Self-rated GenAI knowledge (1–5)	Per 1-point increase	5.4	3.7–7.0	<0.001
Health information ability (0–100)	Per 10-point increase	2	1.2–2.7	<0.001

Sensitivity analyses excluding attention-check failures and unusually rapid submissions yielded similar effect estimates for the primary correlates of preparedness, supporting robustness to these quality flags. The recruitment-pathway sensitivity analysis did not materially change the direction or interpretation of the main associations. Detailed sensitivity estimates should be reported in a supplementary table after verification against the locked analysis file.

Reference categories: age 18–45, sex male, residence urban, education senior secondary/technical, time since diagnosis 1–3 years, treatment status post-operative recovery/follow-up, daily internet use 1–2 h/day, and internet health information use sometimes. Beta coefficients represent adjusted mean differences in preparedness (0–100 scale) unless otherwise specified.

## 4 Discussion

This multi-center, online-only survey provides an early profile of preparedness for GenAI adoption among digitally reachable cancer survivors in Sichuan. Overall preparedness was moderate, with a mean score of 57.8 on a 0–100 scale and 51.3% endorsing near-term intention to try GenAI for health information tasks. Acceptance was use-case dependent. Willingness was higher for using GenAI to help explain test reports than for seeking symptom advice, indicating that participants distinguished between lower-risk comprehension support and decision-adjacent symptom guidance. Preparedness was lower among adults older than 60 years and higher among respondents reporting prior GenAI use and greater self-rated GenAI knowledge. Health information ability also showed a positive independent association with preparedness in adjusted models. These data directly show gradients by task, age, GenAI exposure, and information ability; explanations involving confidence, appraisal capacity, and perceived safety guardrails should be interpreted as plausible mechanisms requiring further testing.

A moderate readiness level in an online-only sample is itself informative. Web-based recruitment tends to select individuals with higher digital access and greater comfort with online platforms, which commonly elevates measured readiness for novel technologies (47, 48). In that context, a preparedness mean below 60 suggests that readiness for GenAI in survivorship is not a simple proxy for smartphone ownership or internet exposure. Instead, the result is consistent with a benefit–risk balancing process in which perceived usefulness coexists with uncertainty about safety and a lack of clear guidance for appropriate use (49, 50).

This sampling feature is central to interpretation. The study estimates preparedness among survivors who could be reached and could complete an online survey; it should not be read as a population estimate for all cancer survivors, especially those with limited smartphone access, limited confidence using WeChat or web forms, or lower health information capacity.

The domain pattern aligns with established technology acceptance theory. UTAUT and the consumer extension UTAUT2 emphasize performance expectancy and effort expectancy as key drivers of behavioral intention, while facilitating conditions support adoption by reducing practical barriers and uncertainty (51, 52). The study pattern, relatively favorable usefulness and ease-of-use perceptions alongside weaker perceived access to guidance or support, fits a context where survivors can envision benefit but remain unsure how to use GenAI safely in a health setting (53). This supports framing preparedness as more than “tech enthusiasm.” It represents a readiness state that depends on whether survivors feel supported in risk management, privacy protection, and appropriate escalation to clinicians (54, 55).

The task-specific willingness gradient supports a risk-stratified view of patient-facing GenAI. Higher willingness for report explanation suggests that survivors conceptualize GenAI as a comprehension aid for technical language, a summarization tool, and a way to prepare questions for follow-up visits, functions that can be verified during later encounters (56). Lower willingness for symptom advice indicates caution when outputs may influence timing of care seeking, medication decisions, or self-triage. This gradient aligns with patient safety concerns around large language models generating fluent but incorrect medical content and the broader need for governance frameworks that match LLM capabilities and limitations (57–59). In practical survivorship care, the gradient implies that implementation should prioritize lower-risk informational supports first, while decision-adjacent functions require stronger safeguards and clearer clinician-facing escalation pathways (60, 61).

The lower willingness for symptom advice should therefore be interpreted as an observed adoption gradient rather than proof that participants correctly classified all high-risk and low-risk tasks. Future work should test whether survivors can reliably distinguish explanation, triage, and treatment-decision functions when presented with realistic GenAI outputs.

Privacy concern functioned as a meaningful readiness constraint. Even among participants willing to complete an online questionnaire, concern about disclosing health information remained moderate. Oncology introduces additional sensitivity because cancer history can be stigmatizing and narratives about recurrence, adverse effects, fertility, sexual health, and psychological distress can carry perceived consequences beyond clinical care (62). Privacy concern also interacts with platform realities, since survivors may access GenAI through commercial services with variable and sometimes unclear data-handling practices (63, 64). Interpreting the finding in public health terms, privacy concern can reduce uptake among precisely the subgroups that might benefit from improved informational access (65). At the same time, the concern is clinically rational, and it should be treated as a legitimate barrier that requires transparent, patient-facing guidance on what to avoid entering, how to anonymize prompts, and when to rely on clinician communication instead of conversational tools (45, 66).

Age disparities were substantial and persisted after adjustment. Adults older than 60 scored about 8 points lower than adults aged 18–45 despite near-universal smartphone access in the sample. This pattern is consistent with a second-level digital divide, where barriers arise from self-efficacy, familiarity with conversational interfaces, and ability to evaluate uncertainty rather than connectivity alone (67, 68). Older survivors may also have higher perceived downside risk from misinformation and may be less comfortable iterating on prompts or cross-checking outputs (69). From an implementation standpoint, the age gradient suggests that survivorship programs should avoid assuming that access implies readiness. Readiness-building for older adults may require simpler onboarding, stronger emphasis on verification habits, and clinician-endorsed framing that positions GenAI as a support for understanding and question preparation rather than a substitute for clinical advice (70, 71).

The data identify an age gradient but do not establish why older survivors reported lower preparedness. Lower interface familiarity, greater perceived risk, more cautious health decision-making, and cohort differences in digital confidence are plausible explanations, but each requires direct testing.

Health information ability was independently associated with preparedness, with a 10-point increase on the 0–100 index corresponding to a 2-point increase in preparedness. The effect size is modest but conceptually important. In practical terms, GenAI use requires survivors not only to ask questions, but also to judge whether an answer is complete, appropriately qualified, and consistent with their own diagnosis, treatment status, and clinician advice. Because LLM outputs are fluent, confident, and conversational, survivors with lower information appraisal skills may be especially vulnerable to over-relying on outputs that appear credible but are incomplete, overly general, or incorrect. This risk differs from conventional searching because conversational responses can reduce the user’s tendency to cross-check sources or notice uncertainty. The association therefore supports a public health interpretation that GenAI may amplify existing disparities in information appraisal and safe-use capacity. Survivors with stronger appraisal skills may be better positioned to use GenAI as a complement to clinical care, whereas survivors with weaker skills may be at greater risk of over-trust, delayed help-seeking, or inappropriate self-management based on AI-generated advice (72–75).

Because the index combined information appraisal and clinician-consultation preference, the association should be interpreted as a relationship between preparedness and information appraisal/help-seeking orientation rather than as evidence for a single narrow skill domain.

This interpretation has direct implications for implementation. Guidance for survivors should be tailored according to information appraisal capacity rather than assuming a one-size-fits-all model of digital support. For survivors with lower health information ability, patient education should emphasize a small number of concrete safety habits: using GenAI mainly for lower-risk tasks such as explaining unfamiliar terms or preparing questions for appointments, avoiding entry of personally identifiable or highly sensitive information, verifying health claims against clinician guidance or authoritative hospital sources, and treating symptom-related outputs as prompts for clinical contact rather than as stand-alone advice. Health systems could further support safer use by providing simple prompt templates, warning examples of misleading but plausible responses, and explicit escalation rules for red-flag symptoms, medication changes, and recurrence concerns. In this sense, lower preparedness should not be interpreted only as reluctance to adopt technology; it may also reflect appropriate caution in the face of limited appraisal skills and unclear guardrails.

GenAI experience and self-rated GenAI knowledge were the strongest correlates of preparedness. Ever users scored about 9 points higher than never users after adjustment, and each one-point increase in self-rated knowledge corresponded to a 5-point increase in preparedness. Several mechanisms are plausible. Experience can reduce interface uncertainty and demonstrate concrete benefits, increasing intention (76). Experience can also improve calibration by revealing limitations, encouraging safer and more selective use (77). At the same time, cross-sectional data cannot resolve directionality. Higher readiness may drive experimentation, and experimentation may also increase readiness (78). Regardless of causal direction, the association suggests that guided exposure and brief education could be impactful, provided education emphasizes safety and privacy rather than encouraging indiscriminate use (79).

The cross-sectional design also means that the association between prior GenAI use and preparedness may reflect self-selection: survivors who were already interested in GenAI may have been more likely to try it before the survey.

Education and residence showed limited independent association with preparedness once GenAI-specific variables and health information ability were included. Mediation is one plausible explanation: structural factors can operate through literacy and exposure, which then carry most of the predictive signal in adjusted models (80). Selection is another plausible explanation: online-only participation compresses variability in digital disadvantage and can attenuate population-level gradients that would be visible with multimode recruitment (68). Either explanation implies that demographic targeting alone will not be sufficient. Programs that seek equitable benefit should focus on modifiable levers, including literacy-oriented guidance, safe-use training, and clinician-endorsed guardrails (81, 82).

### 4.1 Limitations, implications, and future directions

The non-probability sample recruited through clinical contact, WeChat patient groups, and peer referral likely over-represented digitally engaged survivors and may overestimate preparedness relative to the broader survivorship population, particularly among survivors with lower digital access, lower digital confidence, or weaker links to online patient networks. Online-only administration excluded survivors without stable internet access or sufficient digital capability, limiting generalizability. Because center attribution could not be assigned reliably, the analysis could not account for potential site-level heterogeneity. Cross-sectional data prevent causal inference, particularly for associations involving GenAI experience and self-rated knowledge. The preparedness and health information ability measures were theory-informed and internally coherent but were not fully validated scales; future psychometric work should examine dimensionality, item functioning, and measurement invariance. Despite limitations, the results have practical implications: survivorship services can treat preparedness as a modifiable target, prioritize lower-risk GenAI uses such as report explanation and question preparation, and deploy guidance emphasizing verification, privacy protection, and escalation to clinicians when symptoms or treatment decisions are involved.

Future work should validate readiness measures in broader survivorship samples using multimode recruitment, including offline pathways, and should test measurement invariance across age and education strata. Longitudinal designs can evaluate whether baseline preparedness predicts sustained GenAI use and whether guided exposure improves readiness while reducing unsafe reliance. Intervention studies can test brief safety training modules that cover prompt formulation, verification behaviors, privacy-protective prompting, recognition of potentially misleading AI responses, and explicit triggers for clinician contact, particularly for survivors with lower information appraisal capacity. Comparative evaluations of tool features, including source citation, uncertainty communication, and default safety warnings, can clarify which design choices promote safer and more equitable use in survivorship contexts.

## 5 Conclusion

Among digitally reachable cancer survivors in Sichuan, preparedness for GenAI adoption was moderate and varied meaningfully across individuals and tasks. Participants expressed greater willingness to use GenAI for understanding test reports than for symptom advice, supporting a risk-stratified approach to implementation. Older age was associated with lower preparedness even with high device access, suggesting that readiness may depend on confidence and appraisal capacity rather than connectivity alone. Prior GenAI experience and self-rated knowledge were strong correlates of preparedness, and health information ability showed an independent positive association. Because the study used online-only non-probability sampling, the findings should not be generalized to all Chinese cancer survivors without caution. Future survivorship-oriented GenAI integration should prioritize privacy protection, verification habits, and clear escalation to clinicians, with particular attention to older survivors and survivors with lower information appraisal capacity.

## Data Availability

The raw data supporting the conclusions of this article will be made available by the authors, without undue reservation.
